# Oleuropein Attenuates Lipopolysaccharide-Induced Acute Kidney Injury *In Vitro* and *In Vivo* by Regulating Toll-Like Receptor 4 Dimerization

**DOI:** 10.3389/fphar.2021.617314

**Published:** 2021-03-24

**Authors:** Yushun Cui, Hongwei Gao, Shan Han, Renyikun Yuan, Jia He, Youqiong Zhuo, Yu-Lin Feng, Meiwen Tang, Jianfang Feng, Shilin Yang

**Affiliations:** ^1^ College of Pharmacy, Guangxi University of Chinese Medicine, Nanning, China; ^2^ College of Pharmacy, Jiangxi University of Traditional Chinese Medicine, Nanchang, China; ^3^ Guangxi Engineering Technology Research Center of Advantage Chinese Patent Drug and Ethnic Drug Development, Nanning, China; ^4^ State Key Laboratory of Innovative Drug and Efficient Energy-Saving Pharmaceutical Equipment, Nanchang, China

**Keywords:** oleuropein, acute kidney injury, TLR4, NF-κB, MAPK

## Abstract

Acute kidney injury (AKI) is a common critical illness that involves multiple systems and multiple organs with a rapid decline in kidney function over short period. It has a high mortality rate and presents a great treatment challenge for physicians. Oleuropein, the main active constituent of *Ilex pubescens* Hook. et Arn. var. kwangsiensis Hand.-Mazz. displays significant anti-inflammatory activity, although oleuropein’s therapeutic effect and mechanism of action in AKI remain to be elucidated. The present study aimed to further clarify the mechanism by which oleuropein exerts effects on inflammation *in vitro* and *in vivo*. *In vitro*, the inflammatory effect and mechanism were investigated through ELISA, Western blotting, the thermal shift assay, co-immunoprecipitation, and immunofluorescence staining. Lipopolysaccharide (LPS) induced acute kidney injury was employed in an animal model to investigate oleuropein’s therapeutic effect on AKI and mechanism *in vivo*. The underlying mechanisms were investigated by Western blot analysis of kidney tissue. In LPS-stimulated macrophages, our data demonstrated that oleuropein significantly reduced the expression of inflammatory mediators like NO, IL-6, TNF-α, iNOS, and COX-2. Moreover, oleuropein inhibited NF-κB/p65 translocation, and had a negative regulatory effect on key proteins in the NF-κB and MAPK pathways. In addition, the thermal shift and co-immunoprecipitation assays revealed that oleuropein played an essential role in binding to the active sites of TLR4, as well as inhibiting TLR4 dimerization and suppressing the binding of TLR4 to MyD88. Oleuropein markedly alleviated LPS induced acute kidney injury, decreased serum creatinine and blood urea nitrogen (BUN) levels and proinflammatory cytokines. More importantly, the TLR4-MyD88-NF-κB/MAPK pathways were confirmed to play an important role in the oleuropein treatment of AKI. In this study, oleuropein exhibited excellent anti-inflammatory effects by regulating TLR4-MyD88-NF-κB/MAPK axis *in vitro* and *in vivo*, suggesting oleuropein as a candidate molecule for treating AKI.

## Introduction

Acute kidney injury (AKI) is a clinical syndrome caused by a variety of etiologies and pathological mechanisms (Ronco et al., 2019; Jentzer et al., 2020). It is an acute and critical kidney disease, with an characterized by the increasing incidence and high fatality rate (Uchino et al., 2010). Sepsis is a systemic inflammatory response syndrome that can lead to multiple organ dysfunction (Fleischmann et al., 2016), It is the most common cause of AKI(Bagshaw et al., 2008; Hoste et al., 2015). The incidence of AKI in patients with sepsis is 40–50% (Ma et al., 2019). The presence of sepsis in AKI patients increased mortality by 6–8 times, and the risk of poor long-term prognosis (Neyra et al., 2018).

During the onset of sepsis, circulating pathogens and associated molecules, such as lipopolysaccharide (LPS), can be recognized not only by immune system cells, but also by Toll-like receptors (TLRs), pattern recognition receptor on renal tubular epithelial cells. Among the 15 TLR members, TLR4 is the key signaling receptor of LPS (Kang et al., 2009; Balic et al., 2020). When activated by LPS, the TLR4 dimer interacts with the downstream adaptor protein MyD88 to induce MAPK and NF-κB signal transduction (Lu et al., 2017; Hu et al., 2020). TLR4 interacts with MyD88 and transmit signals to the cells, which promotes the activation of TAK1, leading to the phosphorylation of MAPK and the IKK complex, which causes the signal transduction of MAPK and the NF-κB pathway, and finally triggers the release of inflammatory cytokines, such as TNF-a, IL-1β, and IL-6. (Lu et al., 2017; Ahmad et al., 2019; Yuan et al., 2019).

Oleuropein (OP) is a compound isolated from *Ilex pubescens* Hook. et Arn. var. kwangsiensis Hand.-Mazz. widely used in traditional Chinese medicine for its fever-reducing and detoxifying characteristics. Previous studies showed that OP had significant anti-viral, anti-oxidant and anti-inflammatory activity (Omar, 2010). However, evidence for OP’s therapeutic effect and its mechanism of action in AKI has not been shown. The present study aimed to further clarify the inflammation-regulating mechanisms of oleuropein on inflammation, especially in AKI.

## Experimental Section

### Materials

Oleuropein was isolated from *Ilex pubescens* Hook. et Arn. var. kwangsiensis Hand.-Mazz. in our laboratory. *Ilex pubescens* Hook. et Arn. var. kwangsiensis Hand.-Mazz. (100 kg) were soaked with methanol, extracted with ethyl acetate, eluted with dichloromethane-methanol (90:10), and OP was finally obtained by preparative chromatography. The purity was determined by high-performance liquid chromatography (HPLC) to be over 98%. Dulbecco's modified eagle medium (DMEM), fetal bovine serum (FBS) and 0.25% trypsin-EDTA solution were purchased from Gibco Laboratories (Grand Island, NY, United States). Lipopolysaccharides from *Escherichia coli* O111:B4 (L4391), DAF-FM (#251515) and Griess reagent (#G4410) were purchased from Sigma-Aldrich (St. Louis, MO, United States). The protein A/G magnetic bead kit (#88802), TurboFect transfection reagents (#R0531), and quantitative PCR (qPCR) kits were purchased from Thermo Fisher Scientific (Grand Island, NY, United States). Antibodies against p65 (#8242T), IKKα (#2682), IKKβ (#8943T), IκBα (#4814), phospho-p65 (#3033), phospho-IKKα/IKKβ(#2078), phospho-IκBα (#2859), JNK1/2 (#9252), phospho-JNK1/2 (#9255), ERK1/2 (#4695), phospho-ERK1/2 (#4370), p38 MAPK(9212), phospho-p38 (#4511), TAK1 (#4505), phospho-TAK1 (#9339), MyD88 (#4283) and GAPDH (#5174) were obtained from Cell Signaling Technologies (Beverly, MA, United States). Oligonucleotide primers for TNF-α, IL-6 and GAPDH and ELISA kits were obtained from Invitrogen (Grand Island, NY, United States). HA-TLR4, Flag-TLR4, pAP-1-luc and pRL-TK plasmids were purchased from Addgene (Beijing, China).

### Cell Culture

J774A.1 macrophages were obtained from the BeNa Culture Collection (Jiangsu, China), and HEK293T cells were obtained from the Cell Bank of the Chinese Academy of Sciences (Shanghai, China). The cells were cultured in DMEM and 10% fetal bovine serum at 37°C in 5% CO_2_.

### MTT Assay

J774A.1 (1 × 10^4^ cells/well) were cultured in 96-well plates and treated with OP for 24 h. The MTT:3-(4,5-Dimethylthiazol-2-yl)-2,5-diphenyltetrazolium bromide assay was used to assess cytotoxicity as previously reported (Gao et al., 2015). Briefly, MTT solution (5 mg/ml) was added to each well and incubated for another 4 h at 37°C. The supernatant was removed and the left formazan crystals were dissolved in dimethylsulfoxide (DMSO) (100 μL/well). The absorbance was determined by a microplate reader at 570 nm.

### Determination of Nitric Oxide

J774A.1 cells (5 × 10^5^ cells/well) were cultured and allowed to adhere for 12 h. The cells were pretreated with OP (10, 20, and 40 μM) for 1 h, then treated with LPS (1 μg/ml) for 8 h and finally with DAF-FM (1 μM) for 1 h at 37°C. The fluorescence signal was detected by FACScan flow cytometry.

### Enzyme-Linked Immunosorbent Assay

J774A.1 cells were pretreated with OP (10,20, and 40 μM) for 1 h, then co-treated with OP and LPS (1 μg/ml) for 18 h. The supernatant was collected and cytokine concentrations were measured using ELISA kits following the manufacturer’s instructions.

### Immunofluorescence

The immunofluorescence assay was performed as previously described (Gao et al., 2016). Briefly, J774A.1 cells (2 × 10^5^ cells/dish) were plated in confocal dishes and allowed to adhere for 12 h. The cells were pretreated with OP (40 μM) for 1 h and then co-stimulate the cells with LPS (1 μg/ml) for 2 h. Then, the cells were incubated with primary antibody anti-NF-κB/p65 (1:100) overnight at 4°C. Next, the secondary antibody goat antirabbit Alexa Fluor 568 (1:200) was added and incubated at room temperature for 2 h. Hoechst 33,342 (1 μM) was used to stain the nuclei. Images were taken using a confocal laser microscope (Leica, Wetzlar, Germany).

### Western Blotting

The treated cells were collected and total protein was extracted. Cytoplasmic and nuclear proteins were extracted in accordance with the instructions of the kit manufacturer (Beyond time, Shanghai, China). Sodium dodecyl sulfate-polyacrylamide gel electrophoresis (SDS-PAGE) loading buffer was added to the total protein sample and the mixture was boiled at 97°C for 7 min to denature the proteins. Then perform SDS-PAGE was conducted and the proteins were transferred to polyvinylidene fluoride (PVDF) membranes. After blocking with 5% skimmed milk for 2 h, the membranes were treated with primary and secondary antibodies. The chemiluminescence intensity was visualized using a ChemiDoc™ MP Imaging System (Bio-Rad, Hercules,CA, United States).

### Transient Transfection and Luciferase Assay

HEK293T cells (10^6^ cells/dish) were plated in 10 cm dishes and allowed to adhere for 12 h. TLR4-HA (2 μg) and TLR4-Flag (2 μg) plasmids were co-transfected using TurboFect transfection reagents for 24 h. After treatment with OP (40 μM) for 2 h, the cells were co-treated with LPS (1 μg/ml) for 24 h before harvesting.

J774A.1 cells cultured in 96-well plates overnight were transiently transfected with pAP-1-luc plasmids and pRL-TK plasmid according to the manufacturer’s instructions. After 48 h of transfection, the cells were pretreated with OP (10, 20, and 40 μM) for 1 h and stimulated with LPS (1 μg/ml) for 24 h. The luciferase activity was determined using a Dual-Glo luciferase assay system kit according to the manufacturer’s instructions.

### Co-Immunoprecipitation Assay

The antigen samples were combined with the specific antibody (anti-MyD88 or anti-HA) overnight at 4°C with mixing, and then protein A/G magnetic beads were added for 1 h. The immunoprecipitation products were eluted using SDS-PAGE reducing sample buffer.

### Cellular Thermal Shift Assay

J774A.1 cells were treated with OP (40 μM) for 4 h. The total protein was collected and six equal amount heated at 44, 48, 52, 56, 60 or 64°C for 3 min and finally analyzed by Western blotting.

### Quantitative Real-Time Polymerase Chain Reaction (qRT-Polymerase Chain Reaction) Assay

J774A.1 cells were pretreated with OP (10,20, and 40 μM) for 1 h and then with LPS (1 μg/ml) for 4 h. Total RNA was extracted and 1 μg of RNA sample was analyzed by qRT-PCR. SYBR green was incorporated into the PCR amplification reaction. The oligonucleotide primers used for TNF-α,IL-6 and GAPDH were:

TNF-α-F: TTC​TGT​CTA​CTG​AAC​TTC​GGG​GTG​ATC​GGT​CC,
TNF-α-R: GTA​TGA​GAT​AGC​AAA​TCG​GCT​GAC​GGT​GTG​GG,
IL-6-F: TCC​AGT​TGC​CTT​CTT​GGG​AC,
IL-6-R: GTG​TAA​TTA​AGC​CTC​CGA​CTT​G,
GAPDH-F: TGC​CTC​CTG​CAC​CAC​CAA​CT,
GAPDH-R: CCC​CGT​TCA​GCT​CAG​GGA​TGA.

### Animal Experiments and Ethical Statement

The experiments using male BALB/c mice (18–22 g) were approved by the Hunan SJA Laboratory Animal Co., Ltd. (Hunan, China). The mice were housed under specific pathogen-free (SPF) conditions. All animal care and experimental procedures were approved by the Guangxi University of Chinese Medicine Animal Policy and Welfare Committee.

The mice were randomly divided into six groups (12 mice per group): vehicle control, LPS-induced AKI group (2 mg kg^−1^, LPS injected intraperitoneally, i.p.), positive control dexamethasone treatment of the LPS-induced AKI group (5 mg kg^−1^, dexamethasone injected i.p.), OP treatment of the LPS-induced AKI group (10 mg kg^−1^, 20 mg kg^−1^ and 40 mg kg^−1^ of OP injected i.p.). LPS induction of the AKI model was performed as previously described (Plotnikov et al., 2018; Ni et al., 2020). The control mice were given sterile saline according to body weight. In the positive control group, the mice were given dexamethasone after AKI induction. The OP group mice were given OP immediately after AKI induction. The same concentration of OP was also given 12 h after AKI induction. The mice were euthanized by cervical dislocation after 24 h of LPS treatment. Then blood and kidney tissue samples were collected and used for cytokine detection. Part of the kidney tissue was used for histological analysis. The remaining kidney tissue was used for immunoblotting.

### Kidney Index

The kidneys were obtained and weighed, and the kidney index of the mice was calculated as:

kidneyindex(%)=kidneyweight(g)/bodyweight(g)×100

### Histology

The kidney tissue was fixed with 4% paraformaldehyde for 48 h, then washed overnight with running water, dehydrated with ethanol, embedded in paraffin and sliced. Hematoxylin and eosin were used to stain lung tissue.

### Data Analysis

The experimental data are presented as the mean ± SD from at least three independent experiments. GraphPad 6.0 software was used for the statistical analyses. When comparing more than two sets of data, the experimental data were analyzed by the one-way analysis of variance (one-way ANOVA) followed by Dunnett’s multiple comparisons test. A *p*-value of < 0.05 indicated a significant difference.

## Results

### Oleuropein Decreases Inflammation in Macrophages

**FIGURE 1:**
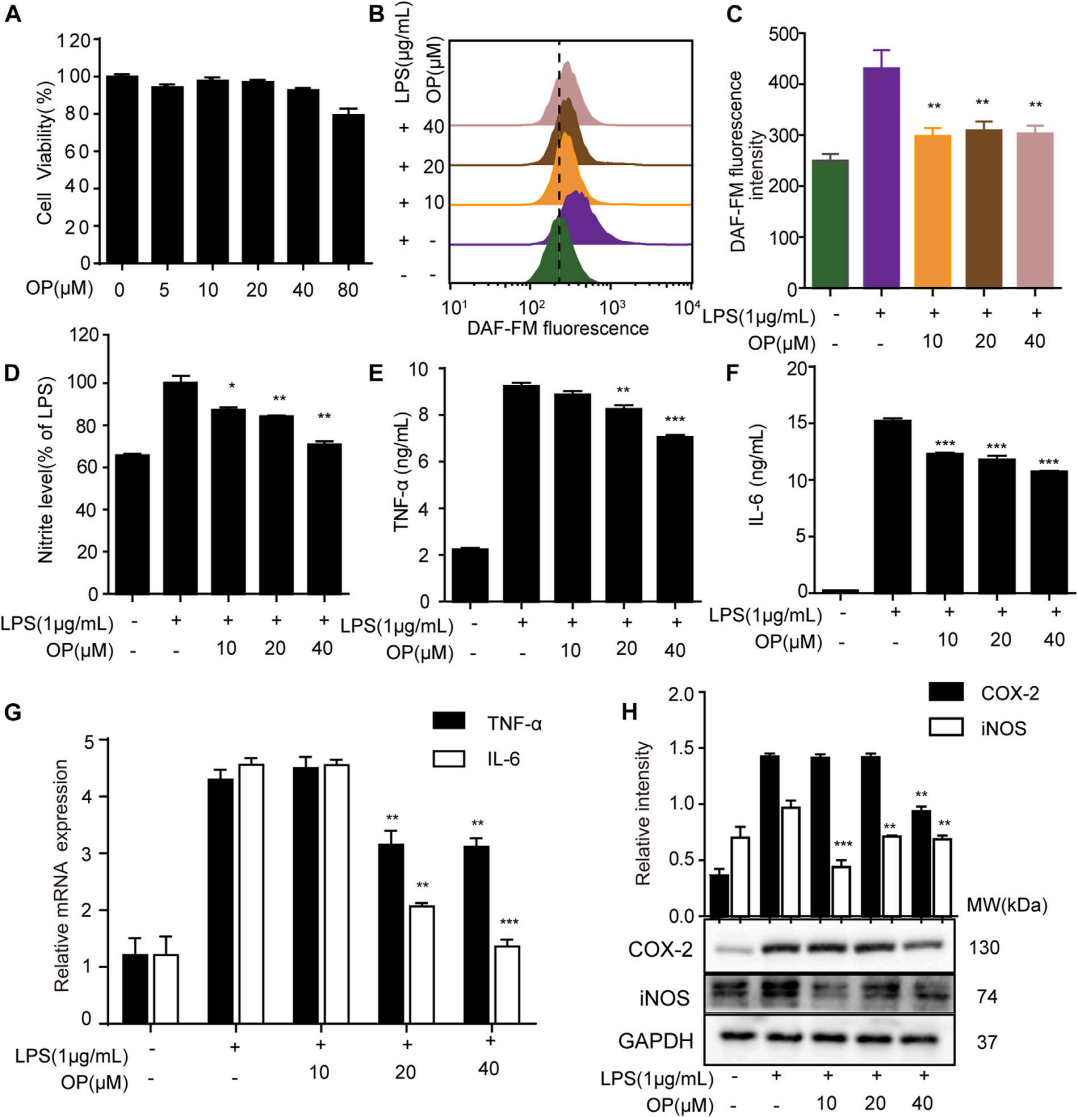
OP relieves inflammation in LPS-induced J774A.1 cells. **(A)** J774A.1 cells were treated with OP for 24 h, and cell viability was determined by the MTT assay. **(B)** The cells were treated with DAFM-DA (1 μM) for 30 min and the NO level was evaluated by flow cytometry. **(C)** Fluorescence statistical analysis of NO. **(D)** Griess assay was used to measure the nitrite levels. **(E,F)** The expression of TNF-α and IL-6 were measured by ELISA. **(G)** The expression of TNF-α and IL-6 were measured by qRT-PCR. **(H)** iNOS and COX-2 levels were determined by Western blot assay. *p < 0.05, **p < 0.01, ***p < 0.001 vs. the LPS alone group.

In LPS-induced macrophage, the overproduction of inflammatory factors such as nitric oxide (NO), nitrite, IL-6, IL-1β, TNF-α, and the overexpression of inflammation-related proteins such as iNOS and COX-2 are important manifestations of inflammation (Singh et al., 2020; Zhang et al., 2020). Here, the effects of OP on LPS-stimulated J774A.1 cells were initially investigated. OP was no obviously toxic to J774A.1 cells at concentrations of 5–40 μΜ (Figure 1A), but significantly reduced NO production (Figures 1B,C). The Griess assay revealed similar results, that OP reduced the excessive increase of nitrite in LPS induced-J774A.1 cells (Figure 1D). In addition, OP significantly reduced the inflammatory factors TNF-a and IL-6 increased by LPS treatment (Figures 1E,F). At the same time, the qRT-PCR experiments obtained similar results at the gene expression level (Figure 1G). Furthermore, Figure 1F shows that OP significantly inhibited the expression of iNOS and COX-2 proteins induced by LPS. Taken together, these results indicated that OP had obvious anti-inflammatory activity.

### Oleuropein Inhibits NF-κB/p65 Nuclear Translocation

**FIGURE 2:**
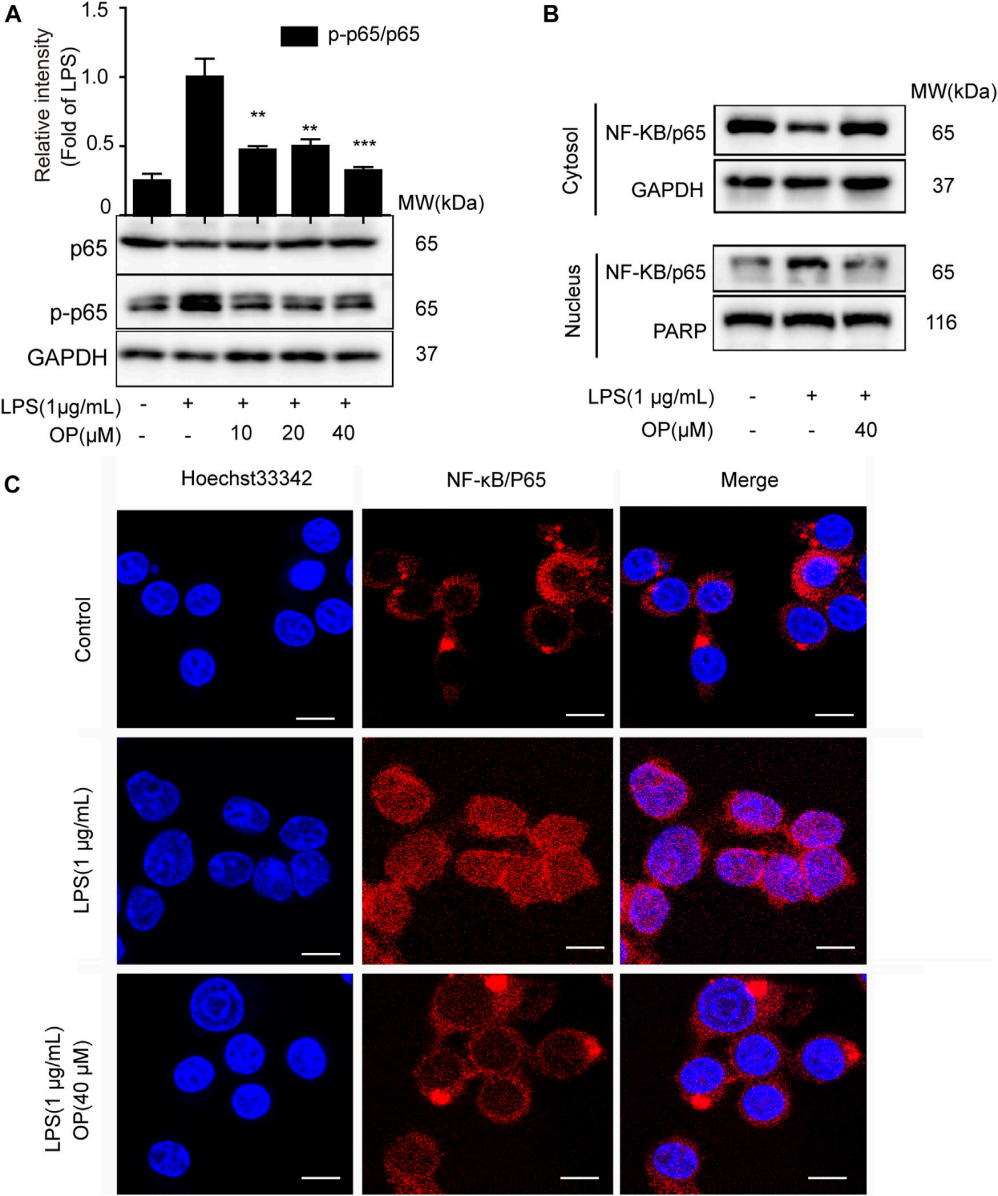
OP inhibits NF-κB nuclear translocation. J774A.1 cells were treated with LPS (1 μg ml^−1^) for 2 h, after pretreatement with OP for 1 h. **(A)**Western blotting was used to analyze NF-κB/p65 and p-p65 expression. **(B)** Cytoplasmic and nuclear proteins in the J774A.1cells were isolated and detected by immunoblot analysis. **(C)** The immunofluorescence assay was applied to detect p65 translocation (scale bar = 10 μm). **p < 0.01, ***p < 0.001 vs. the LPS-alone group.

NF-κB/p65 nuclear translocation plays a central role in the NF-κB signaling pathway (Kong et al., 2020). When p65 is transferred to the nucleus, a variety of proinflammatory factors, chemokines and inflammation-related enzymes are transcribed and expressed, leading to inflammation (Ni et al., 2020). Figure 2A shows that OP suppressed NF-κB/p65 activation in J774A.1 cells. Furthermore, changes in the cytoplasmic and nuclear NF-κB/p65 expression indicated that OP inhibited the LPS-induced translocation of NF-κB/p65 (Figure 2B), which was consistent with the results of the immunofluorescence experiment (Figure 2C). Overall, the results suggested that OP blocked NF-κB p65 nuclear translocation.

### Oleuropein Exerts Anti-inflammatory Effect on NF-κB and Mitogen-Activated Protein Kinase Pathway

**FIGURE 3:**
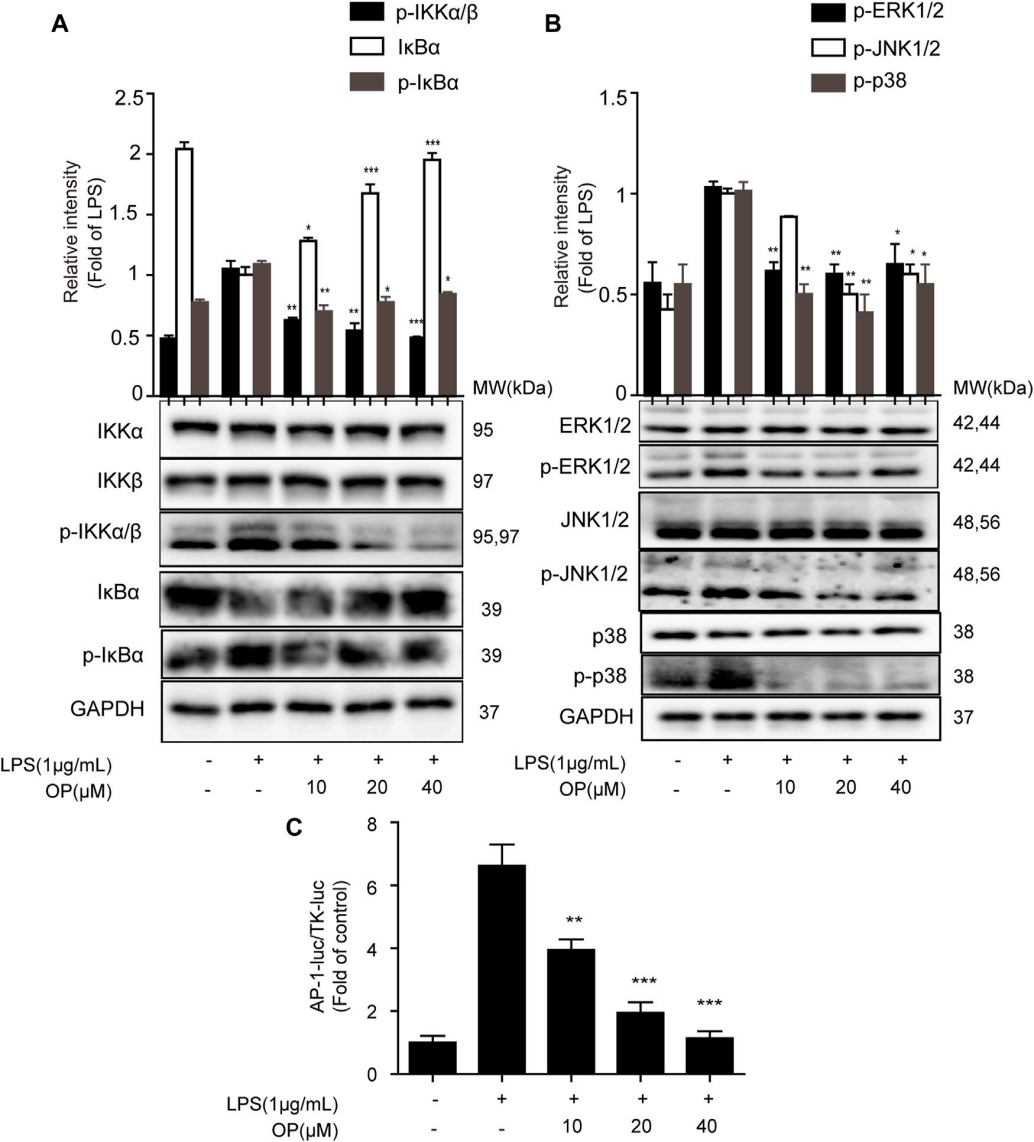
The NF-κB and MAPK pathways were involved in OP's anti-inflammatory effect. J774A.1 cells were treated with LPS (1 μg ml^−1^) for 4 h, after pretreatment with OP for 1 h. **(A)** NF-κB pathway related proteins IκBα, IKKα and IKKβ were detected by Western blotting. **(B)** MAPK pathway related proteins JNK1/2, ERK1/2 and p38 MAPK were detected by Western blotting. **(C)**J774A.1 cells were transiently transfected with AP-1-luc and TK-luc for 48 h. The cells were pretreated with OP(10, 20, 40 μM) before being stimulated with LPS for another 24 h. The luciferase activity was determined using the Dual-Glo luciferase assay. *p < 0.05, **p < 0.01, ***p < 0.001 vs. the LPS-alone group.

To further explore the effect of OP on the NF-κB signal pathway, the expression of IκBα, IKKα and IKKβ protein was analyzed. Figure 3A shows that OP inhibited the degradation of IκBα protein induced by LPS. In addition, the increase in IκBα and IKKα/β phosphorylation induced by LPS could be reversed by OP treatment. The results also showed that OP could reduce the phosphorylation of JNK1/2, ERK1/2 and p38 MAPK induced by LPS, but had no effect on total JNK1/2, ERK1/2 and p38 MAPK protein (Figure 3B) The Dual-Glo luciferase assay was used to evaluate the effects of OP on LPS-induced AP-1 activation in J774A.1 cells. The results showed that OP significantly suppressed LPS-induced AP-1 activation (Figure 3C).

### Toll-Like Receptors 4-MyD88 Signaling was Involved in Oleuropein’s Anti-inflammatory Process

**FIGURE 4:**
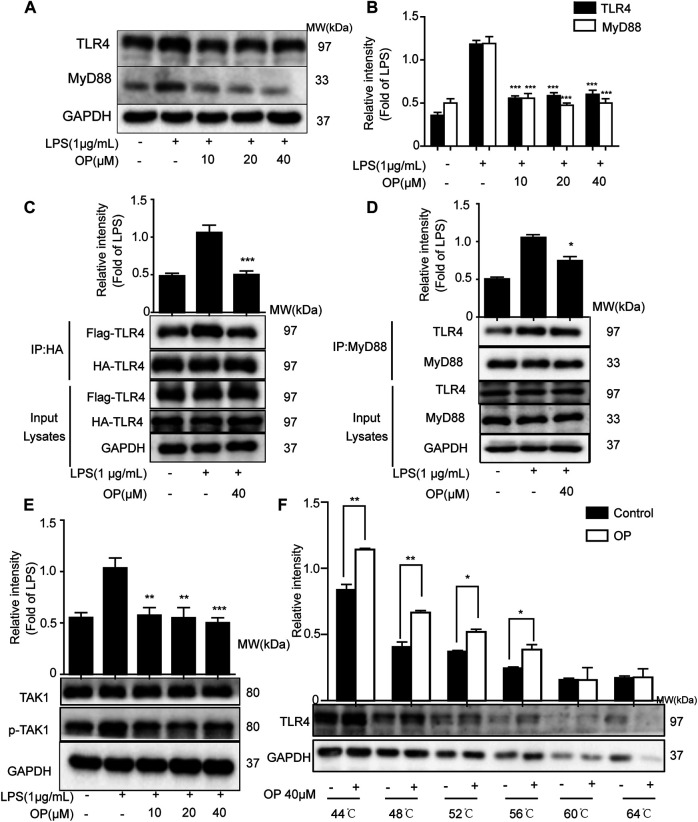
OP suppressed TLR4 dimerization and the MyD88 pathway. J774A.1 cells were treated with LPS (1 μg ml^−1^) for 4 h, after pretreatment with OP for 1 h. **(A)** TLR4 and MyD88 were detected by Western blotting. **(B)** Statistical analysis of TLR4 and MyD88 results. **(C)** HEK293T cells were pretreated with OP (40 μM) for 1 h after transfection with TLR4-Flag and TLR4-HA plasmid for 24 h. Then, the cells were treated with LPS (1 μg ml^−1^) for another 24 h. The proteins were isolated and immunoprecipitated with an antibody against HA. **(D)** Proteins were isolated from J774A.1 cells and immunoprecipitated with an antibody against MyD88. **(E)** J774A.1 cells were treated with LPS (1 μg ml^−1^) for 4 h, after pretreatment with OP for 1 h. TAK1 and *p*-TAK1 were detected by Western blotting. **(F)** J774A.1 cells were treated with OP(40 μM) for 4 h, then the proteins were collected and equal amounts were heated at 44, 48, 52, 56, 60, or 64°C for 3 min. TLR4 was analyzed by Western blotting. *p < 0.05, **p < 0.01, ***p < 0.001 vs. the LPS-alone group.

LPS promotes the dimerization of TLR4 and interacts with the downstream protein MyD88, triggering TAK-1 activation (Ahmad et al., 2019; Hu et al., 2020). Figures 4A,B shows that OP significantly suppressed LPS-induced TLR4 and MyD88 expression. Then a co-immunoprecipitation assay was used to detect the effects of OP on TLR4 dimerization and TLR4-MyD88 complex formation. The results showed that OP suppressed the formation of TLR4 dimers and prevented TLR4 from binding to the downstream adaptor protein MyD88 and forming a complex (Figures 4C,D). In addition, the activation of TAK-1 was alleviated by treatment with OP (Figure 4E). Finally, thermal shift experiments confirmed that OP worked in combination with TLR4 (Figure 4F).

### Oleuropein Ameliorates Lipopolysaccharide-Induced Acute Kidney Injury in Mice

**FIGURE 5:**
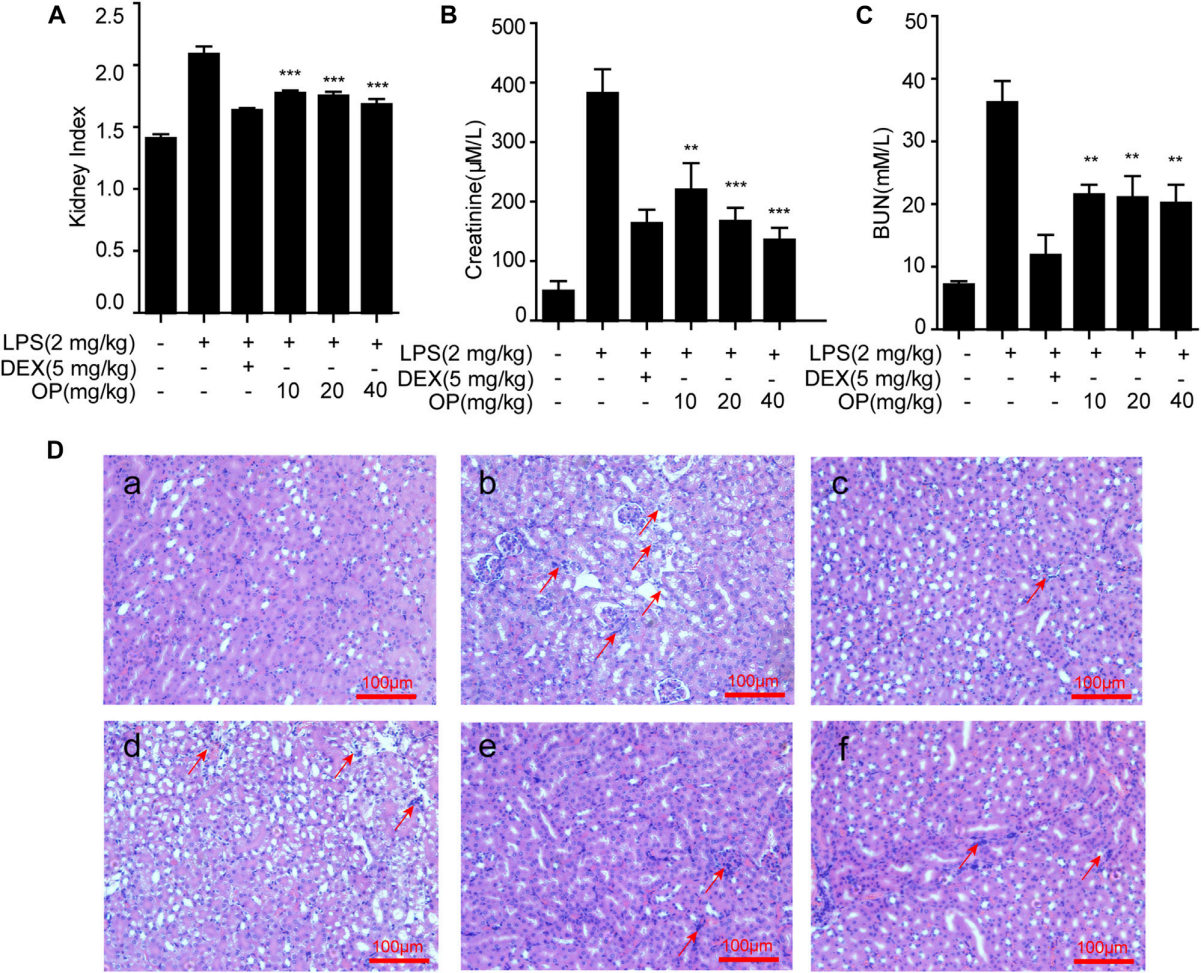
OP ameliorates LPS-induced acute kidney injury. Mice were given OP immediately after LPS injection, and administered OP 12 h after AKI induction. **(A–C)** The kidney index, creatinine and blood urea nitrogen (BUN) were evaluated after LPS induction for 24 h. **(D)** Lung histopathology was assessed via HE staining 24 h after the LPS challenge (200×) Red arrows indicated the lesion or swelling or necrosis or inflammatory infiltration of the kidney tissues. **p < 0.01, ***p < 0.001 vs. the LPS-alone group.

To confirm the therapeutic effects of OP on AKI, mice were treated with LPS by intraperitoneal injection, and the renal index, serum creatinine and blood urea nitrogen (BUN) were detected, and kidney tissue was stained with hematoxylin and eosin (HE). The results indicated that LPS increased the kidney index and the kidney was damaged and enlarged, which was significantly reversed by OP pretreatment (Figure 5A). After treatment with OP, the excessive increase in urine and creatinine levels induced by LPS were significantly alleviated (Figures 5B,C). Furthermore, the histological analysis indicated that LPS triggered the swelling and deformation of renal tubular epithelial cells, and destroyed the normal kidney tissue structure; OP treatment prevented LPS-induced acute kidney injury (Figure 5D).

### Oleuropein Suppresses the Release of Pro-inflammatory Cytokines in Acute Kidney Injury Mice

**FIGURE 6:**
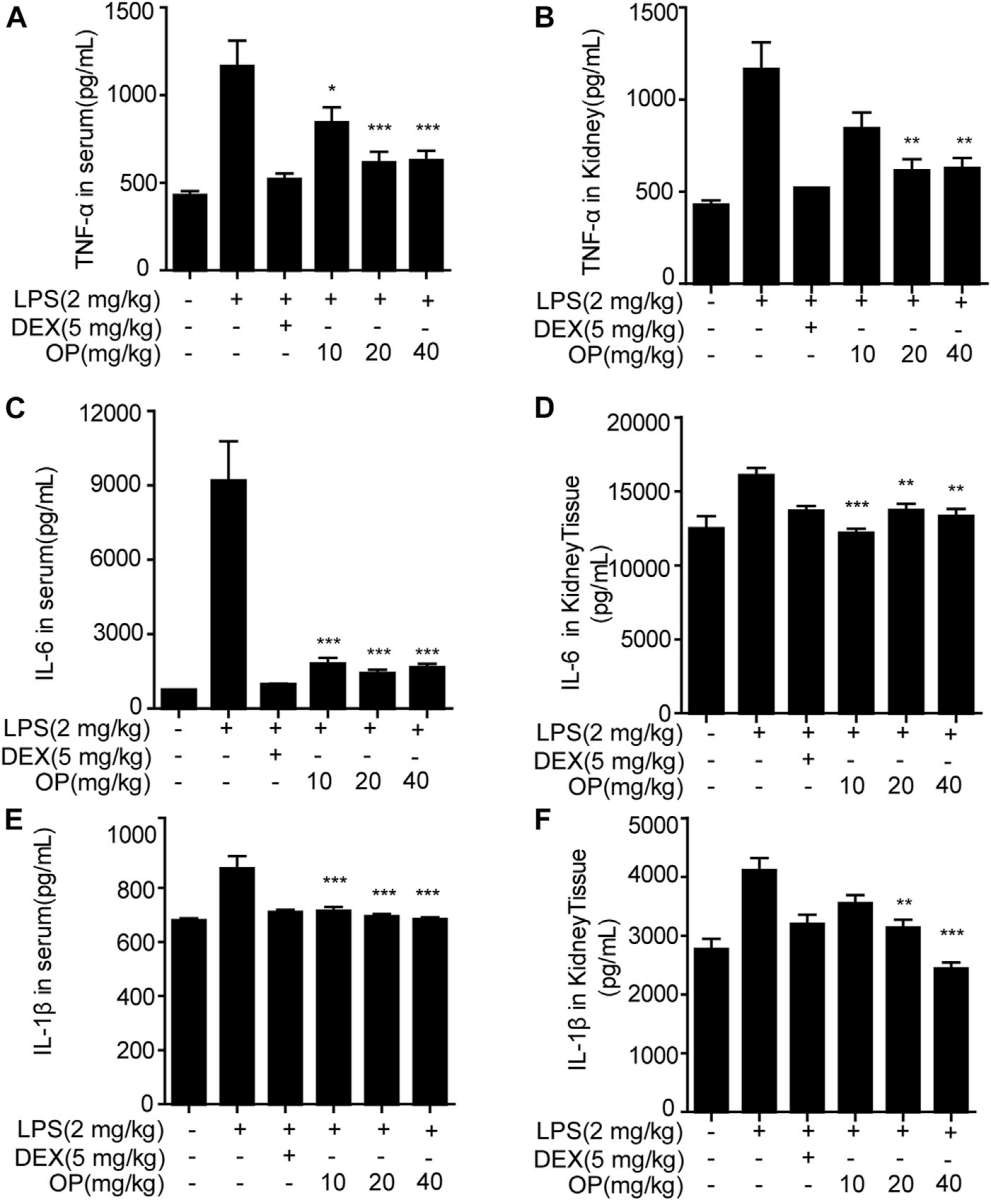
OP inhibits the production of inflammatory factors in AKI mice. Mice were given OP immediately after LPS injection, and administered OP 12 h after AKI induction. TNF-α, IL-6, and IL-1β in serum **(A,C,E)** and kidney tissue **(B,D,F)** were measured using ELISA kits. *p < 0.05, **p < 0.01, ***p < 0.001 vs. the LPS-alone group.

Pro-inflammatory factors, such as TNF-α, IL-6 and IL-1β, play a key roles in the aggravation of AKI (Liang et al., 2017; Ye et al., 2017). In this study, measurements of TNF-α, IL-6 and IL-1β in the serum and kidney tissue of AKI mice were employed. OP significantly alleviated the excessive secretion of inflammatory factors TNF-α (Figure 6A), IL-6 (Figure 6C) and IL-1β (Figure 6E) in the serum in LPS-induced AKI mice. Similar results were found in the kidney tissue (Figures 6B,D,F).

### Oleuropein Ameliorates Acute Kidney Injury in Mice by Regulating the Toll-Like Receptors 4-MyD88-NF-κB/Mitogen-Activated Protein Kinase Pathway

**FIGURE 7:**
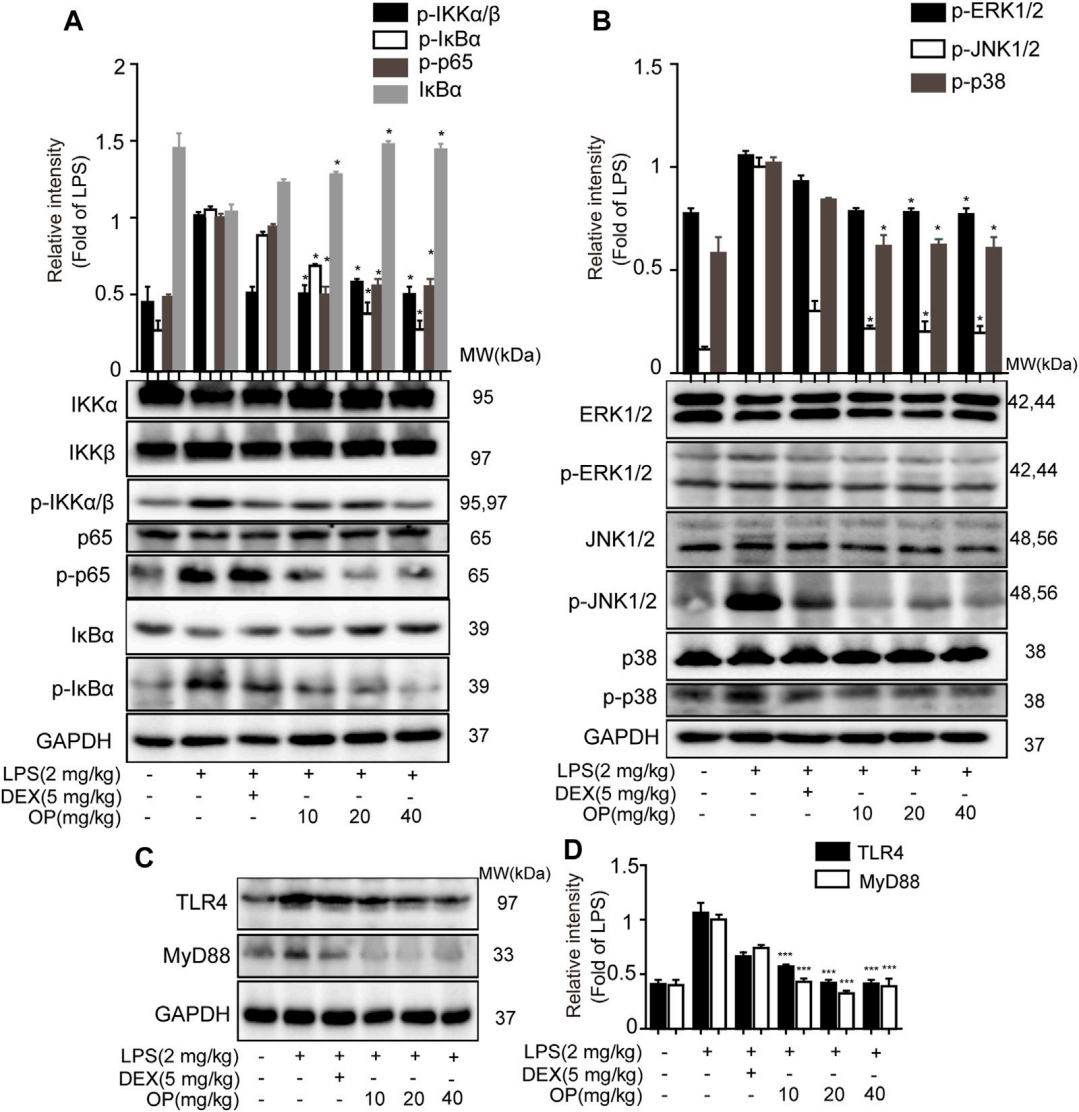
OP protects mice from AKI by regulating the TLR4-MyD88-NF-κB/MAPK pathway. The mice were given OP immediately after LPS injection, and administered OP 12 h after AKI induction. Proteins from the kidneys of AKI mice were isolated 24 h after the LPS challenge. **(A)** NF-κB pathway related proteins p65, IκBα, IKKα and IKKβ were detected by Western blotting. **(B)** MAPK pathway related proteins JNK1/2, ERK1/2 and p38 MAPK were detected by Western blotting. **(C)** TLR4 and MyD88 were detected by Western blotting. **(D)** Statistical analysis of TLR4 and MyD88 results. *p < 0.05, **p < 0.01, ***p < 0.001 vs. the LPS alone group.

For the NF-κB pathway, the core proteins IκBα, p65, IKKα and IKKβ were detected. Significantly, IκBα was activated in AKI mice and the phosphorylation of p65 and IKKα/β increased, which was decreased after OP treatment (Figure 7A). In the MAPK pathway, the results indicated that LPS increased the phosphorylation of JNK1/2, ERK1/2 and p38 MAPK in AKI mice, which was reversed by OP treatment (Figure 7B), suggesting that OP could effectively attenuate activation of the MAPK pathway. Meanwhile, OP pretreated-mice showed less TLR4 and MyD88 expression than those treated with LPS alone (Figures 7C,D). Taken together, our data demonstrated that OP prevented LPS-induced AKI by regulating the TLR4-MyD88-NF-κB/MAPK pathway.

## Discussion

AKI is a widespread and difficult problem worldwide including developing countries (Susantitaphong et al., 2013; James et al., 2020). Although there have been many studies on AKI, there are still no specific treatments (Garcia‐Tsao et al., 2008; Ronco et al., 2019). Sepsis is the main cause of death in critically ill patients, with 35 million new cases of sepsis, and 5.3 million deaths every year (Fleischmann et al., 2016). The pathogenesis of sepsis is mainly related to the inflammatory response, immune dysfunction, and multiple organ failure, in which the kidney is an important target organ (Doi, 2016). Although continuous renal replacement therapy has been actively applied, the mortality rate of AKI patients from sepsis is as high as 70% (Peng et al., 2014). LPS is a type of endotoxin, which can elicit a strong immune response in animals and is widely applied as a pathogenic factor in sepsis research (Remick et al., 2000). Op’s anti-inflammatory activity in LPS-induced macrophages and therapeutic effect in LPS-induced sepsis in AKI was studied.

The overexpression of NO synthase, especially inducible nitric oxide synthase (iNOS), is an important manifestation of LPS-induced macrophages, which leads to the excessive production of NO and eventually inflammation (Yuan et al., 2020). COX-2 plays role similar to that of iNOS, accelerating the release of pro-inflammatory factors TNF-α and IL-6 and mediating the inflammatory response. Oleuropein, a compound classified as secoiridoid, was found in *Ilex pubescens* Hook. et Arn. var. kwangsiensis Hand.-Mazz. and exhibited multiple bioactivities (Omar, 2010). Our results showed that OP could significantly reduce the overexpression of iNOS and COX-2 in J774A.1 cells induced by LPS and inhibit the production of NO, TNF-α and IL-6, thus exhibiting an anti-inflammatory effect.

To further illustrate how OP exerted an anti-inflammatory effect in LPS-induced macrophages, the classic NF-κB and MAPK inflammatory pathways were studied. The results from this study indicated that OP suppressed the transfer of NF-κB/p65 into the nucleus and regulated the key proteins of the NF-κB and MAPK pathway. TLR4 is the receptor for LPS (Tapping et al., 2000; Hajjar et al., 2002). When LPS binds to TLR4, a TLR4 dimer is formed and binds to MyD88 protein, thereby activating the downstream NF-κB and MAPK signaling pathways (Zhou et al., 2018; Gao et al., 2020). Then, the effects of OP on TLR4 dimerization and the related signaling pathways were studied. An immunoprecipitation assay was used to verify the relationship between OP and TLR4. The results indicated that OP inhibited TLR4 dimerization and suppressed the binding of TLR4 to MyD88 protein. In addition, the thermal shift assay revealed that OP could directly bind to TLR4 protein. Taken together, these results suggest that OP exerted anti-inflammatory effects by regulating TLR4 dimer.

Creatinine and urea nitrogen are indicators of renal function and important indicators for clinical diagnosis of AKI (Vaidya et al., 2008). In this study, OP suppressed the increase in creatinine and urea nitrogen in AKI mice. Combined with the results of HE staining, we infered that OP could significantly protect mice from kidney damage. AKI in sepsis is accompanied by systemic inflammation and the excessive production of pro-inflammatory cytokines including TNF-α, IL-6, and IL-1β (Verma and Molitoris, 2015; Al-Harbi et al., 2019). Our results indicated that OP decreased TNF-α, IL-6 and IL-1β in serum and kidney tissue. Taken together, these findings suggested OP as a potential target molecule for the treatment of AKI. The important question was, what are the molecular mechanisms by which OP exerts therapeutic effects in AKI? In the present study, the NF-κB and MAPK pathways were confirmed to be related to OP’s therapeutic effect in AKI mice, consistent with the results of the *in vitro* experiments. Additionally, TLR4 and MyD88 participated in OP’s therapeutic effect. In summary, OP prevented LPS-induced AKI by regulating the TLR4-MyD88-NF-κB/MAPK axis.

## Conclusion

**FIGURE 8:**
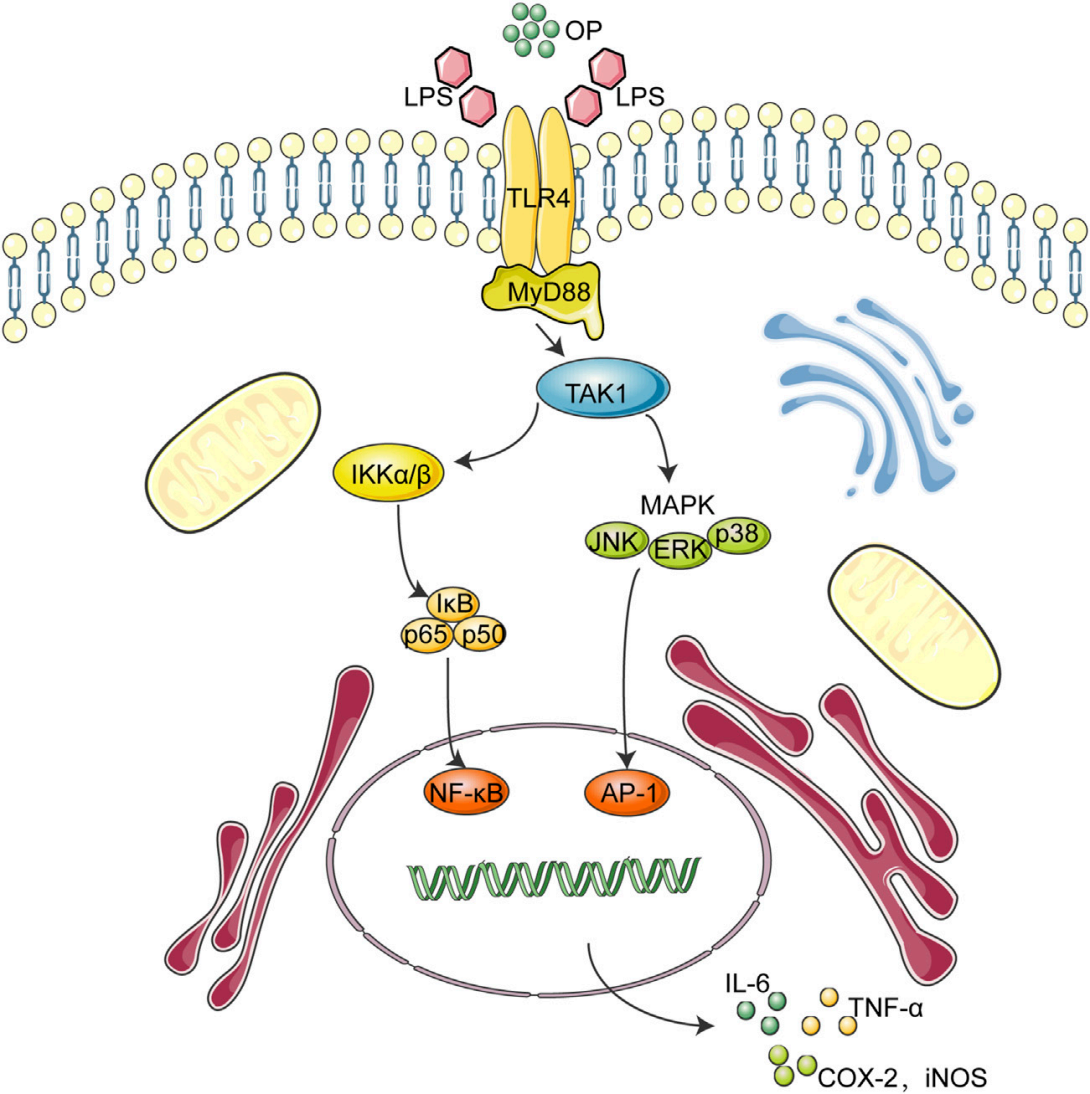
Working model for the OP-based therapeutic effect of AKI.

Overall, the above findings indicated that OP exerted anti-inflammatory effects via NF-κB/MAPK signaling by suppressing TLR4 dimerization. These OP effects may be involved in its ability to ameliorate LPS-associated AKI by regulating the TLR4-MyD88-NF-κB/MAPK axis (Figure 8). Taken together, these findings suggest that OP could be developed as a new molecule for treating AKI.

## Data Availability

The original contributions presented in the study are included in the Supplementary Material, further inquiries can be directed to the corresponding authors.

## Abbreviations

AKI, acute kidney injury; COX-2, cyclooxygenase-2; ERK, extracellular regulated protein kinases; LPS, lipopolysaccharide; JNK, c-Jun N-terminal kinase; LPS, lipopolysaccharides; MAPK, mitogen-activated protein kinase; MyD88, myeloid differentiation primary response gene 88; TAK1, transforming growth factor-beta (TGF-β)-activated kinase 1; TLR, toll-like receptor.
