# Supplementary material for: Oleuropein Attenuates Lipopolysaccharide-Induced Acute Kidney Injury In Vitro and In Vivo by Regulating Toll-Like Receptor 4 Dimerization
Source: Front Pharmacol. 2021 Mar 24;12:617314. doi: 10.3389/fphar.2021.617314 (PMC8024564; doi:10.3389/fphar.2021.617314)
Supplement: Supplementary file 1 [file datasheet1.pdf]

Oleuropein attenuates LPS-induced acute kidney injury *in vitro* and *in vivo* by regulating TLR4 dimerization

Additional Information

The purity of OP was determined by high-performance liquid chromatography (HPLC) to be 98.4.

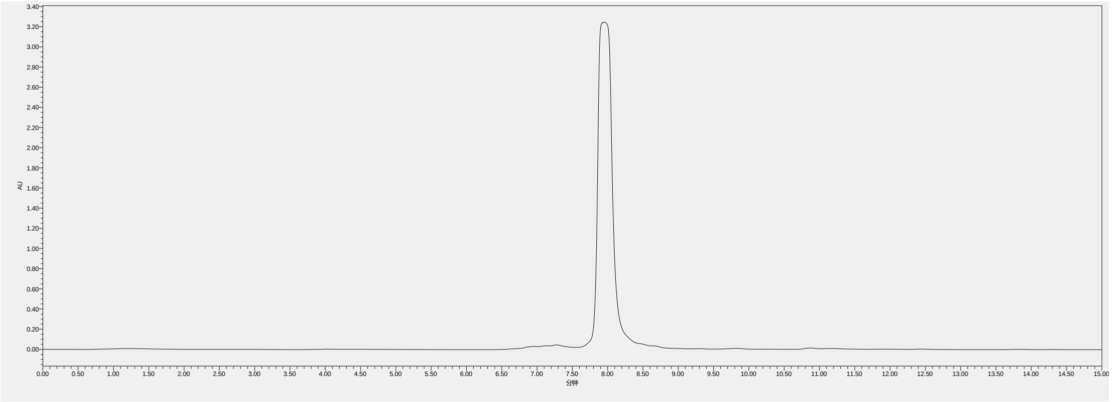

Fig 1 The purity of OP was determined by HPLC.

There is no interference of endogenous endotoxin in OP

LAL test has been performed to exclude the interference of endogenous endotoxin. The results showed that there was no difference in the content of endotoxin between OP and negative control(NC).

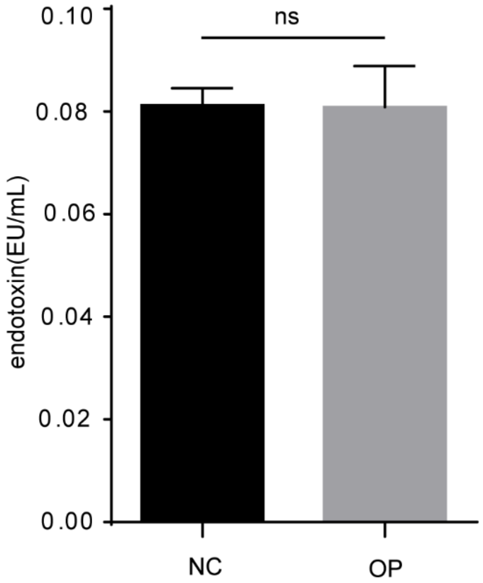

Fig2 There is no interference of endogenous endotoxin in OP. Endotoxin concentrations

were measured using LAL test following the manufacturer's instructions (BIOENDO, EC32545S).

OP has no toxic effect on mice.

Twenty mice were randomly divided into two groups: vehicle control group and drug alone treatment group (40 mg/kg). The control mice were given sterile saline and the drug alone treatment group mice were given OP (40 mg/kg, i.p.). After 12 hours, the two groups of mice were given sterile saline and OP, respectively. The mice were euthanized by cervical dislocation after 24 h of OP treatment. TNF-α, IL-6 and IL-1β, serum creatinine and blood urea nitrogen (BUN) were measured following the manufacturer's instructions The results showed that there was no difference in the content of TNF-α (Fig3A, B), IL-6 (Fig3C,D) and IL-1β (Fig3E,F), serum creatinine(Fig3G) and BUN(Fig3H) between OP alone treatment group(OP) and control group(CON).

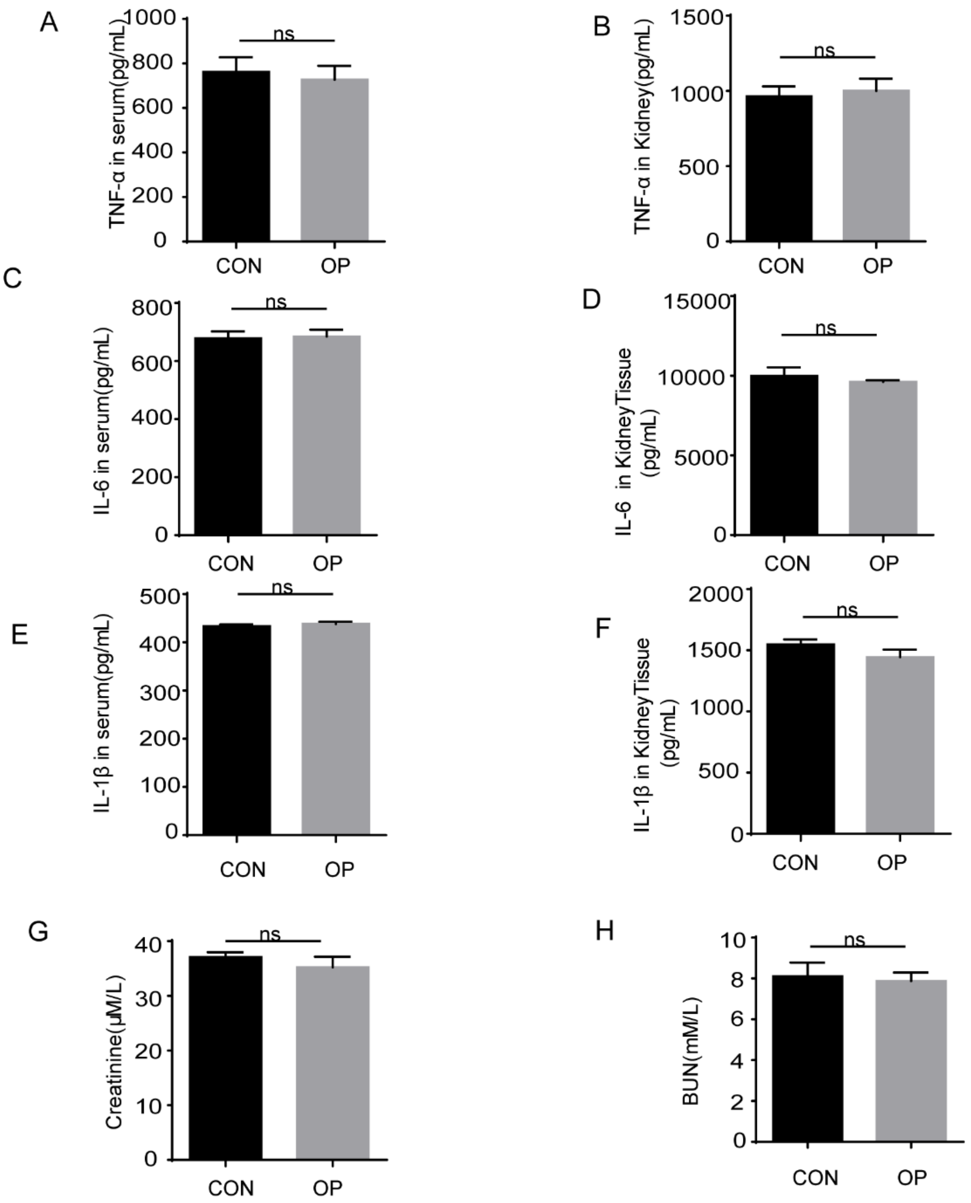

Fig3 OP has no toxic effect on mice. TNF-α, IL-6, and IL-1β in serum (A, C, E) and kidney tissue (B, D, F) were measured using ELISA kits. Serum creatinine(G) and blood urea nitrogen (H) were measured using LAL test following the manufacturer's instructions.
